# PCL-ZnO/TiO_2_/HAp Electrospun Composite Fibers with Applications in Tissue Engineering

**DOI:** 10.3390/polym11111793

**Published:** 2019-11-01

**Authors:** Sorin-Ion Jinga, Andreea-Ioana Zamfirescu, Georgeta Voicu, Monica Enculescu, Alexandru Evanghelidis, Cristina Busuioc

**Affiliations:** 1 Department of Science and Engineering of Oxide Materials and Nanomaterials, Politehnica University of Bucharest, RO-011061 Bucharest, Romania; sorinionjinga@yahoo.com (S.-I.J.); zamfirescu.andreea96@gmail.com (A.-I.Z.); getav2001@yahoo.co.uk (G.V.); 2 Laboratory of Multifunctional Materials and Structures, National Institute of Materials Physics, RO-077125 Magurele, Romania; mdatcu@infim.ro (M.E.); alex.evanghelidis@infim.ro (A.E.)

**Keywords:** fibers, composites, scaffolds, electrospinning, tissue engineering

## Abstract

The main objective of the tissue engineering field is to regenerate the damaged parts of the body by developing biological substitutes that maintain, restore, or improve original tissue function. In this context, by using the electrospinning technique, composite scaffolds based on polycaprolactone (PCL) and inorganic powders were successfully obtained, namely: zinc oxide (ZnO), titanium dioxide (TiO_2_) and hydroxyapatite (HAp). The novelty of this approach consists in the production of fibrous membranes based on a biodegradable polymer and loaded with different types of mineral powders, each of them having a particular function in the resulting composite. Subsequently, the precursor powders and the resulting composite materials were characterized by the structural and morphological point of view in order to determine their applicability in the field of bone regeneration. The biological assays demonstrated that the obtained scaffolds represent support that is accepted by the cell cultures. Through simulated body fluid immersion, the biodegradability of the composites was highlighted, with fiber fragmentation and surface degradation within the testing period.

## 1. Introduction

Diseases, wounds, and traumas can lead to the damage and degeneration of tissues in the human body, which require treatments to facilitate their repair, replacement, or regeneration [1]. Alternatively to the transplantation procedure, tissue engineering aims to heal the affected parts by developing biological substituents that restore, maintain, or improve the original functionality [2,3]. Usually, this field is based on the use of porous three-dimensional scaffolds so that to provide a suitable environment for cell adhesion, proliferation, and differentiation [4,5,6]. The biomimetic concept [6] was adopted for most scaffolds design, in terms of physicochemical properties, as well as bioactivity for superior tissue regeneration. A variety of scaffolds with appropriate features was created by employing different materials, such as polymers, ceramics, and their composites [7,8,9,10].

Among the wide variety of techniques available for producing scaffolds, the electrospinning process is the most commonly approached, showing promising results for tissue engineering applications, including bone reconstruction [11,12,13]. The method is simple and ensures the fabrication of long and continuous fibers, their diameter being possible to control over a wide range, from micrometer to nanometer [14], depending on the processing parameters and optimization. Moreover, the fibrous scaffolds offer a number of advantages, which include high surface to volume ratio, characteristic porous microstructures, and mimicking of the natural extracellular matrix.

The biodegradable materials [15,16] must support the processes of regeneration and repair of bone tissue, while providing mechanical support and, subsequently, degrading into non-toxic products, eventually being eliminated from the body. In the case of synthetic polymers, the most used in the field of hard tissue engineering are polycaprolactone, polylactic acid, polyglycolic acid, and polyethylene glycol [17,18,19,20,21].

Polycaprolactone (PCL) is a semi-crystalline polyester, a biodegradable and biocompatible polymer. The in vivo degradation time for PCL is of approximately two years or more; also, the degradation products are easily assimilated through metabolic pathways, without producing adverse effects [21]. Although the degradable polymeric nanofibers of synthetic or natural origin have been shown to be useful in the growth and proliferation of osteoblasts, ceramics are an appropriate choice for the reconstruction of hard tissues [11]. The advantages of inorganic biomaterials are safety, durability, and stability compared to the organic counterparts [22].

Due to their inherent antibacterial properties, zinc-based nanomaterials [23], especially zinc oxide (ZnO), have attracted the attention of the scientific community, but there are few reports for tissue regeneration applications to date. However, their incorporation into different biomaterials could greatly enhance bone formation [24,25]. Other authors speculated that the composites with titanium dioxide (TiO_2_) have applicability in the biomedical field due to the improvement of cell proliferation, adhesion, and antibacterial properties [26,27,28]. As well, hydroxyapatite (HAp) has been widely used as a bone substitute due to its favorable biological properties, which include biocompatibility, bioactivity, osteoinduction, osteoconduction, as well as osseointegration [29,30]. Composite scaffolds containing hydroxyapatite and biodegradable polymers were manufactured by different research groups in order to obtain a device with applicability in hard tissue engineering [6,31].

In this context, due to the limited range of properties that a polymer possesses, a combination between a polymer and ceramic can lead to the required biological characteristics [25,26,31]. Thus, fibrous composite scaffolds were fabricated by electrospinning, the compositions included PCL and one of the following mineral powders: ZnO, TiO_2_, or HAp. In order to fully understand the relations established between the polymeric phase and inorganic mass, as well to evaluate their behavior and clinic potential correctly, each system was approached in the basic version, namely PCL and only one powder, keeping for future research the complex versions, with several powders considered simultaneously. After the optimization of the experimental conditions, the final samples were evaluated from a compositional and morphological point of view, as well as from the biodegradability and biocompatibility side.

## 2. Materials and Methods

### 2.1. Powders Synthesis

Zinc oxide (ZnO) and titanium dioxide (TiO_2_) were synthesized in the laboratory, using the alkoxide sol-gel protocol and precipitation method, while hydroxyapatite ((Ca_5_(PO_4_)_3_(OH), HAp) powder was purchased from Sigma-Aldrich (Merck KGaA, St. Louis, MO, USA). For the preparation of ZnO powder, zinc acetate dihydrate (Zn(CH_3_COO)_2_·2H_2_O, Sigma-Aldrich, Merck KGaA, St. Louis, MO, USA) was employed as the cation precursor, absolute ethanol (C_2_H_5_OH, Sigma-Aldrich, Merck KGaA, St. Louis, MO, USA) as the solvent, and monoethanolamine (C_2_H_7_NO, MEA, Sigma-Aldrich, Merck KGaA, St. Louis, MO, USA) as synthesis additive; the molar ratio between Zn(CH_3_COO)_2_·2H_2_O and MEA was set at 1:2. After homogenization by magnetic stirring at 60 °C for 2 h, the amount of water necessary for hydrolysis was added, and the system was allowed to jellify. The resulting gel was dried and calcined at 900 °C for 2 h, in air, with a heating rate of 10 °C/min and natural cooling, in order to remove the gas generating components. For the synthesis of TiO_2_, titanium isopropoxide (Ti[(CH_3_)_2_CHO]_4_, Sigma-Aldrich, Merck KGaA, St. Louis, MO, USA) and isopropyl alcohol (C_3_H_8_O) were used as starting materials, distilled water was added for hydrolysis. The obtained precipitate was filtered, washed, dried, and calcined at 400 °C for 2 h, in the same atmosphere and heating/cooling conditions as previously described, so as to ensure the transition from titanium hydroxides or oxyhydroxides to the final oxide.

### 2.2. Fiber Preparation

The composites were prepared from an organic component, to which the inorganic powders were added one at a time. Thus, polycaprolactone ((C_6_H_10_O_2_)*_n_*, 80.000 Da, PCL, Sigma-Aldrich, Merck KGaA, St. Louis, MO, USA) was selected as biodegradable matrix, chloroform (CHCl_3_, CF, Sigma-Aldrich) and *N*,*N*-dimethylformamide (C_3_H_7_NO, DMF, Sigma-Aldrich, Merck KGaA, St. Louis, MO, USA) as solvents, while the electrospinning technique as the procedure for generating one-dimensional structures; the volumetric ratio between CF and DMF was maintained at 4:1.

The precursor solutions for electrospinning were prepared in a two-stage approach. First, a suspension of inorganic powder in the solvent mixture was achieved by dispersing 0.5 g of solid in 10 mL of liquid, all being ultrasonicated for 5 min at 50% amplitude. Then, 1.6 g of polymer was dissolved in the previously prepared suspension, which led to a final solution with 16% PCL and 5% inorganic powder; this was maintained under magnetic stirring for 24 h for the purpose of PCL solubilization and general homogenization.

Each solution was loaded into a 2 mL syringe connected with a stainless steel 21 G needle, having 0.8 mm inner diameter. A direct current high voltage source was necessary to provide an electrostatic field of 15 kV. For the fibers deposition, a static collector was fitted to the equipment, to which glass substrates used as fibers support were attached. The distance between nozzle and collector was set at 25 cm, while the feed rate was 3 mL/h. The electrospinning process was performed at a room temperature of 18 °C and relative humidity of 30%.

### 2.3. SamplesCharacterization

The samples characterization was carried out by X-ray diffraction (XRD) with a Shimadzu XRD 6000 diffractometer (Shimadzu Corporation, Kyoto, Japan) with Ni filtered Cu Kα radiation (*λ* = 0.154 nm), 2*θ* ranging between 20° and 70°; scanning electron microscopy (SEM), coupled with energy-dispersive X-ray spectroscopy (EDX), with a FEI Quanta Inspect F electron microscope (FEI Company, Hillsboro, OR, USA); UV-Visible spectroscopy (UV-Vis) with a PerkinElmer Lambda 45 spectrophotometer (PerkinElmer, Waltham, MA, USA), the wavenumber ranging between 200 and 900 nm; and thermal analysis with a Shimadzu DTG-60 equipment (Shimadzu Corporation, Kyoto, Japan), in the 20–800 °C temperature range, in air.

The biological evaluation was accomplished through *in vitro* tests: simulated body fluid (SBF) immersion for 14 days, at 37 °C, the testing solution being prepared according to Kokubo [32], as well as optical fluorescence microscopy [33], MTT assay [34] and GSH assay [35], on mesenchymal stem cells. The biocompatibility was analyzed in accordance with the law in force and following standard procedures, after samples sterilization under UV irradiation for 30 min. In order to evaluate the cell proliferation and cytotoxicity of the obtained materials, the MTT biochemical assay was employed; this is a colorimetric method based on a reduction process correlated with the enzymatic cell activity, for which the absorbance was read at 570 nm with a Tecan spectrophotometer. The cellular response to oxidative stress was estimated on the basis of GSH assay, the luminescence being recorded with a Titertek–Berthold luminometer; this method indirectly detects and quantifies the amount of an antioxidant agent produced by the cells in different testing environments, giving information about the toxicological response and oxidative stress level. Furthermore, the viability of the cells in the presence of the investigated samples was assessed by fluorescent microscopy, using Red CMTPX fluorophore; the images were taken with a Carl Zeiss digital camera. A detailed description of the working protocols is available in the specification sheets of each testing kit [33,34,35].

## 3. Results and Discussion

### 3.1. Physicochemical Characterization

The inorganic powders were investigated by XRD, SEM, and UV-Vis spectroscopy in order to evaluate their crystalline structure and morphology, as well as bandgap in the case of the two semiconductor oxides (ZnO and TiO_2_).

**Figure 1:**
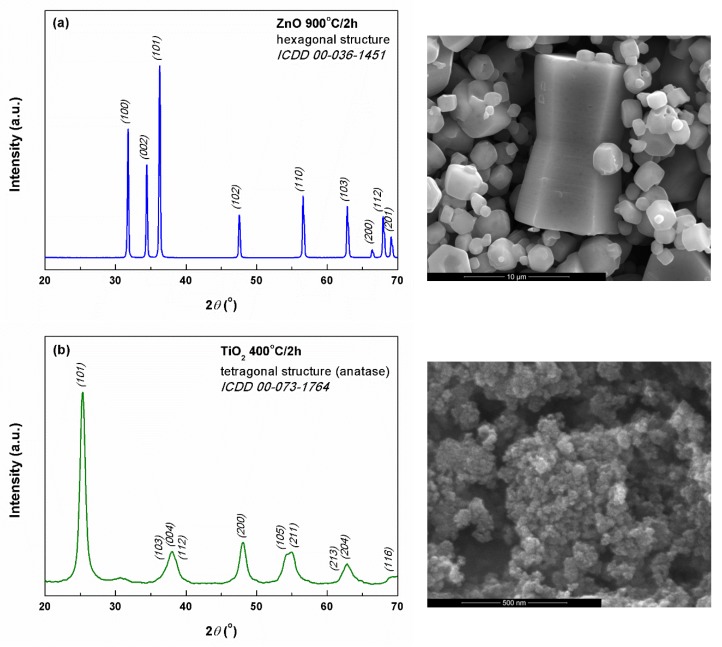

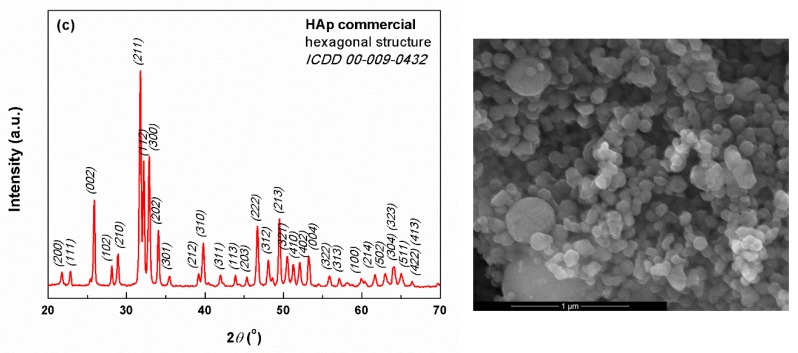
XRD patterns (left) and SEM images (right) of the mineral powders: (**a**) ZnO, (**b**) TiO_2_, and (**c**) HAp.

The XRD pattern of ZnO powder (Figure 1a) contains only the diffraction peaks corresponding to the crystalline planes of wurtzite-type ZnO with hexagonal symmetry. The situation is similar in the case of TiO_2_ powder, for which the XRD pattern shown in Figure 1b indicates the obtaining of anatase-type TiO_2_ with tetragonal symmetry. On another hand, the HAp commercial powder turned out to be highly crystalline and of hexagonal structure (Figure 1c). For all three inorganic materials, no diffraction maxima associated with secondary phases or impurities were distinguished.

Going to the microstructural characterization, the SEM image of ZnO (Figure 1a) shows the existence of at least three families of particles: the first one with complex appearance, resulted by connecting two pyramid trunks with hexagonal base, with dimensions on the longest side of about 10 μm; the second one in the form of polyhedral structures with hexagonal cross-section, containing a cavity, probably the structural units from which the shapes of the first category have resulted; the third represented by ordinary polyhedral particles, with a relatively wide size distribution (from less than 1 to 4 μm). TiO_2_ powder is made up of quasi-spherical particles with diameters below 50 nm and pronounced tendency of agglomeration due to the large specific surface area, as seen in Figure 1b. Also, it can be stated that the size distribution is relatively narrow, which could be an advantage in the subsequent production of composite materials with a high degree of homogeneity. Moving to HAp powder marketed by Sigma-Aldrich, the product data sheet claims the existence of particles with dimensions below 200 nm, an aspect mostly confirmed by the SEM image (Figure 1c). Most of the particles fall in the 50–100 nm size range, their shape being quasi-spherical or slightly faceted.

Using Scherrer’s formula [36], the average crystallite size of all three powders was calculated by mediation on the first three most intense diffraction peaks. The resulting values are as follows: 49 nm for ZnO, 8 nm for TiO_2_, and 40 nm for HAp. As was expected, ZnO presented a larger value than TiO_2_, due to the fact that the calcining temperature was higher and promoted the crystallites development. However, all inorganic masses can be considered nanostructured, and, in this way, the achievement of fibrous composites containing such zero-dimensional structures is favored, as long as it is possible to disaggregate the agglomerations into individual entities. Moreover, the obtained results could be very well correlated with the information provided by the SEM images (Figure 1) in terms of crystallite-particle dimensionality.

Further, by employing the UV-Vis spectra and Kubelka–Munk approach [37], the bandgap of ZnO and TiO_2_ were determined to be around 3.1 eV, slightly lower than those reported in the scientific literature for different similar nanostructures [37,38]. Briefly, using the reflectance data, *F(R)* function was calculated and *(F(R)·E)^1/2^* function was plotted versus photon energy (*E*) in order to graphically estimate the band gap values; Kubelka-Munk function is expressed as *F(R) = (1 − R)^2^/(2R)*, where *R* is the observed diffuse reflectance. Moreover, for the two oxides, the antimicrobial activity against two microbial strains was assessed, namely *Staphylococcus Aureus* (Gram-positive model) and *Escherichia Coli* (Gram-negative model). ZnO displayed an antimicrobial effect on both bacteria, the diameter of the inhibition zones being 8 and 7 mm, respectively. Shortly, the antimicrobial potential was assessed using the agar diffusion test; after 20 min of sterilization under UV irradiation, the powders were mixed with sterile saline solution, from which a defined volume was taken and placed on agar plates inoculated with the microorganism to be tested, the antibacterial effect is quantified by measuring the diameter of the inhibition zone after incubation at 37 °C for 24 h.

**Figure 2:**
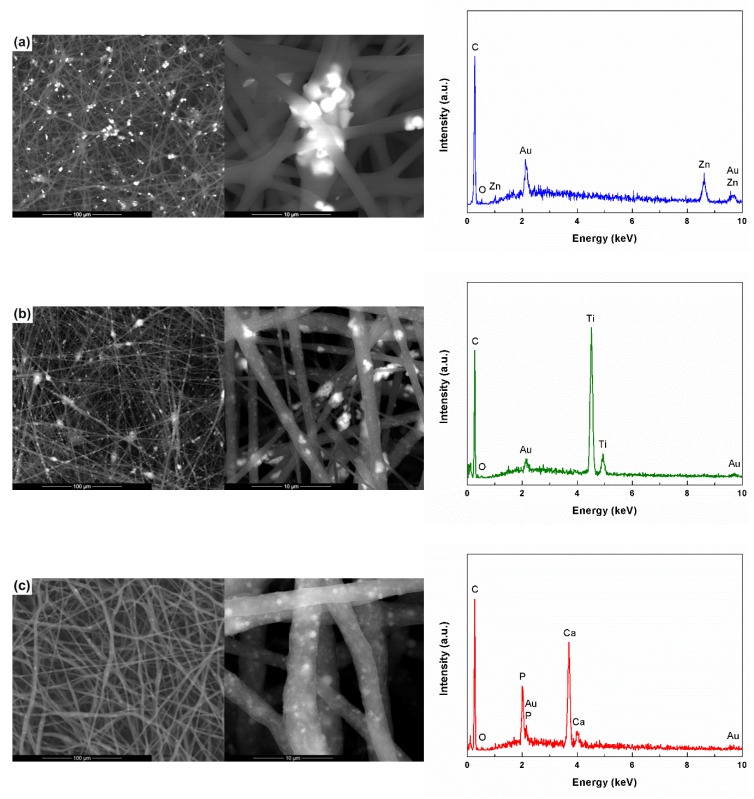

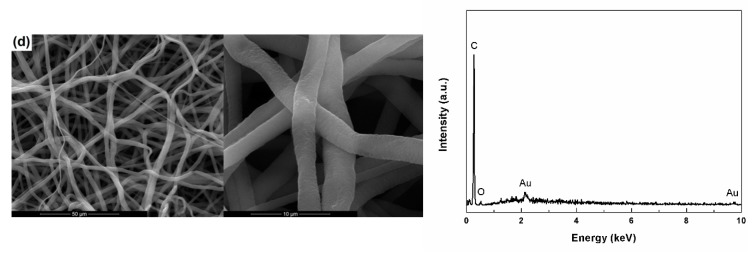
SEM images (left) and EDX spectra (right) of the composite and unitary scaffolds: (**a**) PCL-ZnO, (**b**) PCL-TiO_2_, (**c**) PCL-HAp and (**d**) PCL.

The electrospun composites were first analyzed from the microstructural and compositional point of view, the corresponding images and EDX spectra being exhibited in Figure 2. In order to be able to correctly evaluate the influence of the addition of the inorganic powders on the properties of the PCL fibers, the reference sample, without inorganic content, was also analyzed.

PCL-ZnO composite (Figure 2a) has a quite high homogeneity due to the random distribution of ZnO particles among PCL fibers, mainly near the intersection areas. There is also a certain tendency of agglomeration, with aggregates of particles reaching dimensions up to 10 μm. It should also be emphasized that certain particles are embedded in the polymeric fibers, which leads to surface passivation and reduced sample efficiency in those types of determinations that are based on the active role of the surface. All well, the particle embedding also leads to an increase in the fiber diameter, which normally ranges between 2 and 3 μm.

Regarding PCL-TiO_2_ composite (Figure 2b), the tendency of agglomeration and attachment in the form of aggregates to the polymeric fibers is higher than in the previous case, the particles are this time nanometric in size; this aspect also has a negative effect on the sample homogeneity in large areas. Moreover, the modification of the powder nature influences the diameter of the fibers. fibres diameter, in the sense that the emergence of fibres with much smaller diameters, below 500 nm, is favored.

The third category of composites, PCL-HAp (Figure 2c), has a slightly modified morphology, with the distribution of the inorganic particles predominantly in the volume of the polymeric fibers, at different depths, and less on the surface. The homogeneity is relatively good this time, since the tendency of agglomeration is reduced. The fibers size does not undergo substantial changes with the addition of HAp, maintaining it in the 2–4 μm range.

Goring to the bare fibers (Figure 2d), the SEM image shows a network of one-dimensional polymeric structures, non-woven and randomly distributed in the plane of each layer, the average diameter being of approximately 2 μm. Compared to the composite samples, the flexibility of this fully polymeric sample is higher, a claim supported by the snake-like arrangement of the fibers.

To demonstrate the loading of the fibers with particles of different compositions, EDX spectra were employed. As expected, to the elements specific to PCL (C and O), supplementary signals assigned to the elements of each inorganic powder are added (Zn, Ti, Ca, and P). The peaks of Au are due to the samples preparation protocol for SEM investigation, namely the deposition of a nanometric layer of conductive material on the entire surface.

**Figure 3:**
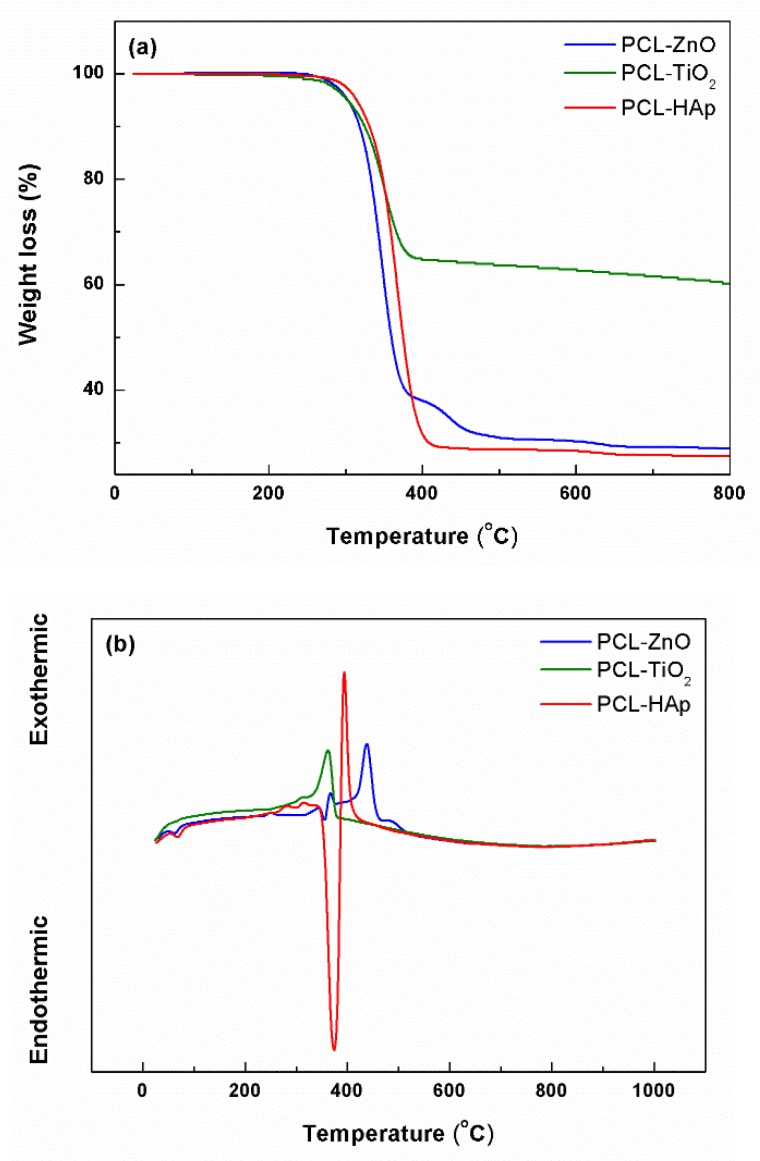
Thermal analyses of the composite scaffolds: (**a**) weight loss, and (**b**) differential thermal analysis.

From the complex thermal analyses performed on the fibrous composite scaffolds and presented in Figure 3, it was found that in the 20–800 °C temperature range, there is a total weight loss of 71% for PCL-ZnO, 40% for PCL-TiO_2_, and 73% for PCL-HAp, respectively. These losses were recorded below the temperature of 400 °C, mainly in the range 250–400 °C, representing 94–99% of the total mass loss. The main loss is always accompanied by an exothermic effect centered between 350 and 450 °C, generated by the combustion of the organic component. However, in the case of PCL-ZnO and PCL-HAp, the weight losses occur in two stages, the first one is endothermic and the second one is exothermic. The shift of the exothermic effect to higher temperatures when ZnO is present is most likely due to the fact that this oxide influences polymer stability. In the case of HAp, the endothermic effect can be correlated with the existence of less crystalline phases within the commercial product, which undergoes a dehydration process.

Given the use of a fixed concentration of inorganic powder, it was expected that the mass loss would be similar in all three situations. However, the differences are significant, both between the three types of composites and the expected value (around 76%). Analyzing comparatively, it can be observed that the losses recorded for the samples containing ZnO and HAp displayed the closest values to the one theoretically calculated, being only a few percent lower, a result that can be explained through the existence of a certain proportion of residual solvents in the fibers. On the other hand, the composite containing TiO_2_ showed a much lower loss, which means that the concentration of inorganic powder in the final sample is higher than the designed one; this behavior can be associated with the stability of the precursor solution, as well as the nanometric size of TiO_2_ particles.

### 3.2. Biological Characterization

**Figure 4:**
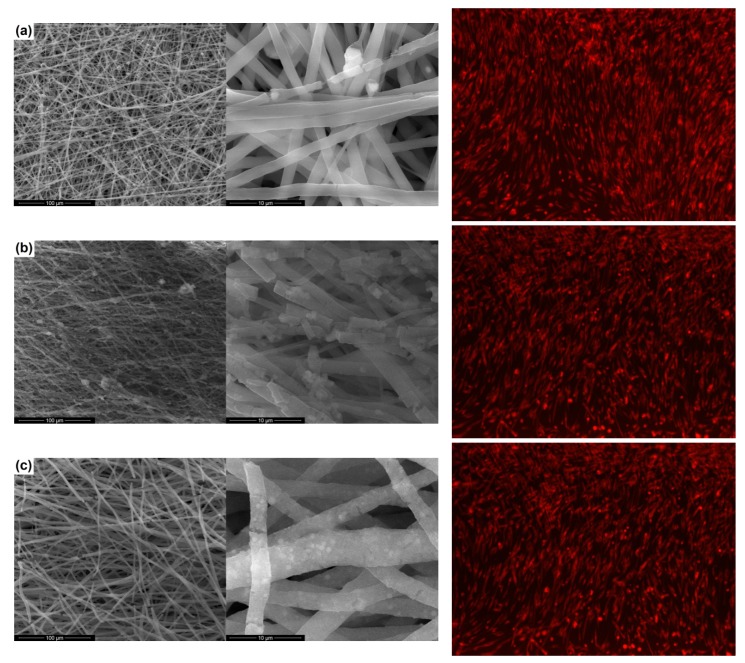
SEM images after SBF immersion for 14 days (left) and fluorescence microscopy images (right) of mesenchymal stem cells in contact with the composite scaffolds: (**a**) PCL-ZnO, (**b**) PCL-TiO_2_, and (**c**) PCL-HAp.

Although the biodegradation time of PCL is well defined, the *in vitro* studies related to the biodegradability determination for the PCL scaffolds obtained by the electrospinning technique is limited [39,40]. Thus, the fibrous scaffolds realized in this work were characterized by the SBF test, so as to assess the behavior when in contact with the physiological environment for 14 days; the biodegradability of the composite materials was revealed through SEM images (Figure 4). A substantial change in the morphology of the fiber during the soaking period can be detected. The one-dimensional structures have multiple breaks, their surface getting a rough appearance, probably due to the chemical attack of the testing solution on PCL; this process involves reactions between the carboxyl groups on the polymeric chains and the cationic species in the testing solution, resulting in by-products that decrease the material stability in the aqueous medium. This aspect confirms that the degradation is triggered on the surface of the fibers and evolves towards the inner regions. Comparatively, the highest tendency to disintegration was observed in the case of the PCL-TiO_2_ sample.

**Figure 5:**
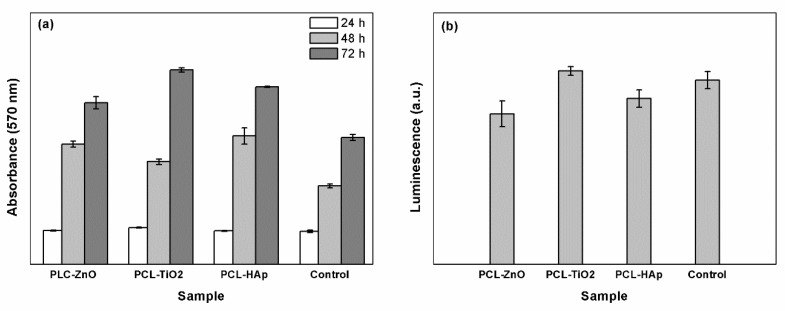
Biological evaluation of the composite scaffolds: (**a**) MTT assay, and (**b**) GSH assay.

The composite fibers obtained by electrospinning were characterized *in vitro* using cellular assays, considering that it has been reported in the scientific literature that a concentration of oxide powder above a certain threshold may have a toxic effect on cells. Cell proliferation was assessed by the MTT assay (Figure 5a), while cell viability was determined by the GSH assay (Figure 5b) in association with optical fluorescence microscopy (Figure 4).

Regarding cell proliferation, the values recorded for the specimens and control were graphically represented in Figure 5a and indicate that the tested materials do not have a cytotoxic effect, the absorbance showing higher values compared to control (differences between 7 and 15%). In other words, a higher intensity of the recorded signal is translated into more metabolically active viable cells and conserved cellular integrity. In all three cases, cell proliferation shows a significant increase from 24 to 72 h; thus, the cell number increases with cell incubation time, which suggests that all composites sustain cell proliferation.

From the point of view of the oxidative stress, the results presented in Figure 5b confirm that the final scaffolds represent supports accepted by the cells. Comparatively, PCL-ZnO and PCL-HAp samples showed the lowest oxidative stress; however, PCL-TiO_2_ exhibited a similar value with the control, but considering that it falls within the limit of errors, it is still a potential candidate for medical applications.

The optical fluorescence microscopy images from Figure 4 confirm the previous results, revealing that the investigated fibers have no cytotoxic effect; the cells are viable and have normal morphology. The cellular viability is also demonstrated by the fact that their metabolism is active, the cells incorporating the dye into the cytoplasm.

## 4. Conclusions

Using the electrospinning technique, composite scaffolds based on polycaprolactone and inorganic powders (zinc oxide, titanium dioxide, and hydroxyapatite) were successfully obtained. The inorganic components were proved to be highly crystalline and composed of particles having dimensions in the nanometric or micrometric field, while the composite materials presented fibers with diameters below 5 μm and a relatively homogenous distribution of the powders within the polymeric fibrous networks. By means of simulated body fluid soaking, the biodegradability of the composite materials was highlighted, noting a considerable tendency of fragmentation and surface degradation during the 14 days of testing. Following the biological tests, it was found that the resulting fibrous composites represent support accepted by the cell cultures, displaying significant cell proliferation. Taking into account both the properties of biodegradability and biocompatibility, it can be concluded that the proposed systems represent candidates with considerable potential in the field of tissue engineering. Future improvements can be achieved by optimizing the processing parameters or by incorporating several types of mineral phases in order to achieve multifunctional composites.
